# The Application of Machine Learning Algorithms to Bond Strength between Steel Rebars and Concrete Using Bayesian Optimization

**DOI:** 10.3390/ma17184641

**Published:** 2024-09-21

**Authors:** Huajun Yan, Nan Xie, Dandan Shen

**Affiliations:** 1 School of Civil Engineering, Beijing Jiaotong University, Beijing 100044, China; n_xie@sina.com; 2 SANY Heavy Industry Co., Ltd., Beijing 100044, China; shendd2@sany.com.cn

**Keywords:** machine learning, Bayesian optimization, bond strength, Shapley additive explanation

## Abstract

The purpose of this study is to estimate the bond strength between steel rebars and concrete using machine learning (ML) algorithms with Bayesian optimization (BO). It is important to conduct beam tests to determine the bond strength since it is affected by stress fields. A machine learning approach for bond strength based on 401 beam tests with six impact factors is presented in this paper. The model is composed of three standard algorithms, including random forest (RF), support vector regression (SVR), and extreme gradient boosting (XGBoost), combined with the BO technique. Compared to empirical models, BO-XGB`oost was found to be the most accurate method, with values of R^2^, MAE, and RMSE of 0.87, 0.897 MPa, and 1.516 MPa for the test set. The development of a simplified model that contains three input variables (diameter of the rebar, yield strength of reinforcement, concrete compressive strength) has been proposed to make it more convenient to apply. According to this prediction, the Shapley additive explanation (SHAP) can help explain why the ML-based model predicts the particular outcome it does. By utilizing machine learning algorithms to predict complex interfacial mechanical behavior, it is possible to improve the accuracy of the model.

## 1. Introduction

Composite actions between steel rebars and concrete affect the mechanics of structures significantly. The effectiveness of an integrated reinforced concrete (RC) structure is determined by the strength of the bond between the steel rebars and concrete. Tests for bond strength have been conducted in experimental studies using several methods, including pull-out tests, beam anchorage tests, beam-end tests, and lap spice tests [1]. A key reason for the popularity of the pull-out test is its simplicity of manufacture. The steel rebars are under tension in the pull-out test specimens, whereas the concrete is under compression. Therefore, it is not appropriate to use pull-out tests to determine bond behavior, as also discouraged in ACI 408R-03 [1]. Due to the tensile nature of RC members, pull-out tests are not suitable for determining bond strength. In general, a beam test is considered a reliable method for assessing bond strength because it is able to provide a description of the bonding state in real-life situations [2,3].

**Figure 1:**
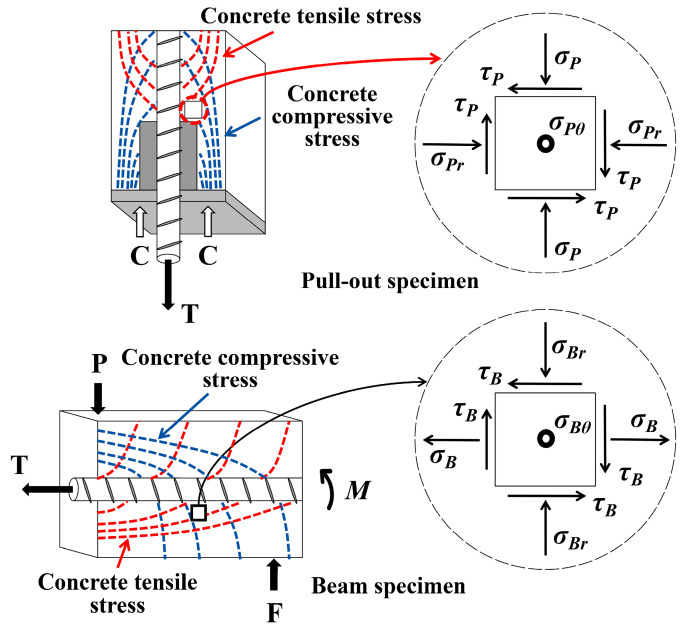
The distribution of stress in the pull-out test and beam test.

Figure 1 illustrates the stress state of the concrete surrounding the steel rebar. In the pull-out test, $\sigma_{P}$ refers to the compressive stress that results from the pressure applied at the end of the specimen. The compression stress and the shear stress are represented by $\sigma_{Pr}$ and $\tau_{P}$, respectively. The $\sigma_{P\theta }$ represents the circumferential tensile stress that results from the compressive force exerted by a steel rebar. Only splitting cracks are observed by $\sigma_{P}$ in the pull-out test specimens, and the bond stress distribution along the rebar is continuous. $\sigma_{B}$ refers to the tensile stress produced during the flexure of a beam specimen during the beam test. The ring compressive stress and shear stress are represented by $\sigma_{Br}$ and $\tau_{B}$, respectively. A steel rebar generates circumferential tensile stress, which is described by $\sigma_{B\theta }$. Rebar stress is redistributed when a crack occurs due to flexure. During crack progression, bond pressure cannot exert enough force on concrete to cause it to split or separate. Assuming the same local bond strength and the same development length, the average bond strength of the beam test should be lower than that of the pull-out test.

The concrete and steel rebar interfacial bonds enable the transfer of forces and the development of composite responses [4]. A combination of three factors contributes to the force transfer between steel rebars and concrete: chemical adhesion, friction, and mechanical interlocking [5]. The latter action is typically dominant when relative slip occurs on ribbed bars. Due to this, the strength of the bond is primarily determined by the steel rebars and the concrete, as well as their stress levels. There are several empirical models available for calculating the bond strength ($\tau_{max}$) [6,7,8,9]. The development length of the ACI 318 code is determined according to a model developed by Orangun et al. [9]. Recent studies by Torre-Casanova et al. [10] have proposed formulas for separating failures caused by splitting from those caused by pull-out failure. Furthermore, mechanical models were proposed as a complement to experimental testing for the evaluation of the interfacial properties [6,7]. In spite of their past success, mechanical models have shown some shortcomings, including assumptions that are not valid under certain conditions [8]. A mechanical model generally considers a small number of influencing factors, since many factors complicate the mechanical model. The high dependence on the database used in the analysis may result in significant errors when applying these equations to different scenarios [11]. For instance, the square root of concrete’s compressive strength is typically used to estimate the bond’s strength [12], but this calculation was found to be inaccurate due to coupling effects [13]. Although empirical formulas are reliable, fracture mechanics methods only take into account a limited number of variables (such as the compressive strength of concrete) [14]. 

As a result of the nonlinearity of the relationship between steel rebars and concrete, the aforementioned empirical models are insufficient to explain the mechanism of bond strength. The development of data-driven models was based on various machine learning (ML) approaches, which have proven to be effective [15,16,17,18,19]. An artificial neural network (ANN) approach was developed by Dahou et al. [20] on the basis of 112 pull-out tests. Furthermore, 117 pull-out tests were used to develop a model that predicts bond strength [21]. As a result of Wang et al.’s study, relevant data have been collected on the bond strength of concrete and corroded steel rebars, a bond strength model has been proposed, and the relationship between various parameters and bond strength has been clarified [22]. With artificial neural network (ANN) models, Mousavi et al. [23] then predict the ultimate and relative bond strength between corroded rebars and the surrounding concrete with and without transverse rebars. Based on the results of these studies, the proposed model was found to be acceptable. However, the bond-slip behavior of steel-concrete in the pull-out test is not indicative of the behavior of real reinforced concrete beams [24]. In light of this, Jeong et al. [25] collected 75 beam specimens and 255 pull-out data and used ML algorithms (random forest and K-means clustering) to analyze the influence of each feature within two groups. Compared to previous equations, the proposed model could fit test results more accurately and with less dispersion.

ML approaches are capable of predicting steel rebar-concrete behavior, however certain challenges still need to be overcome, including the lack of credible beam test data, which makes it difficult to develop a reliable bond strength model using hyperparameter optimized ML algorithms. In structural mechanics, it has always been difficult to combine ML approaches for nonlinear material properties with theoretical knowledge. 

To overcome the limitations of conventional empirical formulas and data-driven models, this study proposes a method for calculating the bond strength between steel rebars and the surrounding concrete using ML approaches. It is possible to enhance the accuracy of the model by using ML algorithms with Bayesian optimization technology. 

Further, complex ML models are difficult to explain, and only a few studies have explored this question [26]. The Shapley additive explanation (SHAP) method simplifies the interpretation of ML models by utilizing game theory [27]. A wide range of fields have been affected by the SHAP, including structural engineering [28], infrastructure systems [29], and material properties of concrete [30]. The use of ML-based models coupled with the SHAP method for bond strength prediction has not been widely adopted. As a result of combining SHAP with ML algorithms, this paper offers vital insights into the complicated nonlinear behavior of bond strength, as well as illustrating how various features contribute to bond strength.

## 2. Methodology

### 2.1. Existing Bond Strength Equations

As shown in Table 1, empirical equations are presented for estimating the ultimate bond strength ($\tau_{u}$). A model proposed by Orangun et al. [9] indicates that bond stress is linearly impacted by the minimum spacing and the thickness of the cover. In contrast, Esfahani’s models are based on beam-end samples, while other equations were derived from beam splice test results [7,31]. Several models are also proposed in the design codes, such as ACI 408R-03 [1] and AS 3600 [32]. 

### 2.2. The Considered Machine Learning (ML) Algorithms

The ML models have been generated using Python 3.7. It is possible to categorize ML algorithms as linear or nonlinear models based on the linear correlation or the complex nonlinear correlation between input variables and outcomes. Several models are selected to train estimators of bond strength, including a linear model named support vector machine regression (SVR) and two nonlinear models named random forest (RF) and extreme gradient boosting (XGBoost). In SVR models, parameters are optimized by maximizing the minimum distance between the support vector and the hyperplane. As opposed to the RF model where individual learners are independent, the XGBoost model is dependent. Therefore, the data-driven models in this study are trained using these three algorithms. This selection was based on the fact that these three models have different mechanisms and typical characteristics in linear models as well as in nonlinear models.

#### 2.2.1. Support Vector Regression (SVR)

SVR is an application for minimizing the structural risk associated with support vector machines [34]. A regression function in hyperspace can be obtained by using the SVR model presented by $f\left(x\right)=\omega^{T}\phi \left(x\right)+b$. By using the loss function, the SVR model is obtained as shown in the following equations:

(1) $$\underset{\omega ,b,\xi_{1,\;}\xi_{2,\;}}{\min}F\left(X\right)=\left(\frac{1}{2}\right){\left\|\omega \right\|}^{2}+C\left(e^{T}{\;\xi }_{1}+e^{T}{\;\xi }_{2}\right)$$

where ω, x, and b are the weight vector, input vector, and bias vector, respectively. In the preceding equations, *C* is the fixed parameter, $\xi_{1}$ and $\xi_{2}$ are dummy parameters, and *e* represents the vector unit.

(2) $$\underset{\alpha ,\;\alpha^{*}}{max}G\left(X\right)=\left(\frac{1}{2}\right){\left(\alpha^{*}-\alpha \right)}^{T}K\left(A,A^{T}\right)\left(\alpha^{*}-\alpha \right)+e^{T}\left(\alpha^{*}+\alpha \right)$$

(3) $$f\left(x\right)=\sum_{i=1}^{n}\left(\alpha^{*}-\alpha \right)K\left(x_{i},x\right)+b$$

where α and $\alpha^{*}$ are the Lagrange multipliers, $K\left(x_{i},x\right)$ is the Kernel function, and *A* is the input for the training set.

#### 2.2.2. Random Forest (RF)

A random forest (RF) is an ensemble learning algorithm that involves the combination of a large number of decision trees [35]. The concept of randomly chosen decision trees was introduced by Tin Kam [36], and Breiman [37] elaborated on it with additional algorithmic parameters. To construct different decision trees and to average their forecasts, the RF utilizes the bootstrap resampling technique. Bootstrap resampling minimizes the correlation between decision trees, improving the algorithm’s generalization accuracy. Various bootstrap samples are used to build each regression tree. The hyperparameters determine the maximum depth and branching of each regression tree.

Combining several decision tree learners using least square boost allows for improved results, as well as reduced variance and overfitting. A major advantage of using trees for bagging is that they are capable of capturing complex interactions in data and have a relatively low level of bias when they are cultivated to a sufficient depth.

#### 2.2.3. Extreme Gradient Boosting (XGBoost)

XGBoost is an advanced ensemble machine learning algorithm that incorporates gradient boosting [38]. Within this framework, XGBoost introduces a number of enhancements, including: (1) As a result of XGBoost, a structural risk term is incorporated into the objective function in order to achieve greater accuracy and avoid overfitting; (2) As part of XGBoost’s moderation process, it samples rows and columns to reduce overfitting; and (3) Through XGBoost, the branching metric is reset, leading to a more direct growth of the decision tree.

The XGBoost algorithm is an ensemble algorithm that calculates the predicted value by combining the values of the decision trees.

(4) $${\hat{Y}}_{i}=\sum_{k=1}^{K}{\alpha_{k}f}_{k}\left(X_{i}\right)$$

where K represents the maximum depth of a tree, ${\hat{Y}}_{i}$ is the predicted value. In a single decision tree, $f_{k}\left(X_{i}\right)$ represents the prediction function, and $\alpha_{k}$ is a learning rate used to prevent overfitting in the training model.

The objective function for the XGBoost algorithm can be expressed as follows:

(5) $$F_{obj}^{t}=\sum_{i=1}^{n}\left(L\left(Y_{i},{{\hat{Y}}_{i}}^{t-1}\right)+f_{t}\left(x_{i}\right)\right)+\Omega \left(f_{t}\right)$$

where an objective function is defined as $F_{obj}^{t}$, *L* represents the loss function, *Y_i_* represents the actual value of the *i*-th sample, and $\Omega \left(f_{t}\right)$ is the regularization terms. 

#### 2.2.4. Bayesian Optimization (BO)

**Figure 2:**
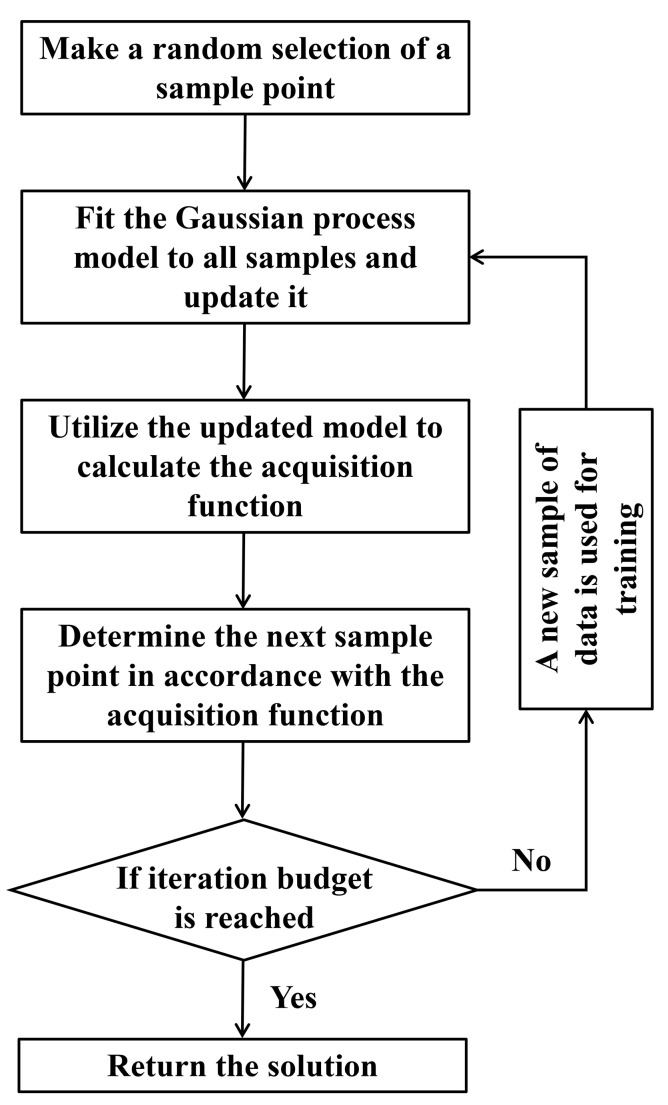
Workflow of Bayesian optimization.

In order to optimize model performance, the most appropriate set of hyperparameters within a particular search space must be chosen. This study selects the most appropriate set of hyperparameters using Bayesian optimization, which is an efficient global optimization technique for complex black-box functions [39]. The workflow of BO is illustrated in Figure 2. 

It is possible to accomplish this by assuming that the objective function is a multivariate Gaussian process (GP). To implement GP, the kernel of the covariance matrix is fitted as follows:

(6) $$K=\left[\begin{array}{ccc} K\left(x_{1},x_{1}\right) & \cdots & K\left(x_{1},x_{n}\right)\\ \vdots & \ddots & \vdots \\ K\left(x_{n},x_{1}\right) & \cdots & K\left(x_{n},x_{n}\right) \end{array}\right]+\sigma_{noise}^{2}$$

where GP is calculated using n sample points of aging creep; K represents the covariance kernel, which is calculated as an exponential function of the second norm of the difference between two samples; and in normal distribution, $\sigma_{noise}$ represents the standard deviation of the noise [40].

In order to estimate the improving potential of every possible sample point, an acquisition function is calculated by expected improvement (EI) as follows:

(7) $$I\left(x_{n}\right)=\left(y_{best}-\mu \left(x_{n}\right)\right)\Phi \left(\frac{y_{best}-\mu \left(x_{n}\right)}{\sigma \left(x_{n}\right)}\right)+\sigma \left(x_{n}\right)\phi \left(\frac{y_{best}-\mu \left(x_{n}\right)}{\sigma \left(x_{n}\right)}\right)$$

where Φ and ϕ represent the standard normal density and distribution functions, respectively; $y_{best}$ represents a tentative optimal value.

### 2.3. Evaluation Metrics

In order to evaluate ML-based models effectively, it is imperative to select metrics that are accurate reflections of their performance. A total of three evaluation metrics were selected as part of this study, namely the coefficient of determination (R^2^), the root mean square error (RMSE), and the mean absolute error (MAE). A model is generally considered to perform better if R^2^ is close to 1. The MAE and RMSE values are all within $\left[0,\right.\left.+\infty \right)$ and the smaller values indicate that the predicted value is closer to the true value, thus indicating that the model is more accurate. The formulas are as follow:

(8) $$R^{2}=1-\frac{\sum_{i=1}^{n}{\left(y_{pred,i}-y_{exp,i}\right)}^{2}}{\sum_{i=1}^{n}{\left(y_{pred,i}-\frac{1}{n}\sum_{i=1}^{n}y_{exp,i}\right)}^{2}}$$

(9) $$RMSE=\sqrt{\frac{1}{n}\sum_{i=1}^{n}{\left(y_{pred,i}-y_{exp,i}\right)}^{2}}$$

(10) $$MAE=\frac{1}{n}\sum_{i=1}^{n}\left|y_{pred,i}-y_{exp,i}\right|$$

where the value of $y_{pred,i}$ and $y_{exp,i}$ are the predicted value and actual value, respectively.

## 3. Model Development

### 3.1. Database for Beam Tests

The bond strength was calculated using ML algorithms based on a regression analysis of existing beam test specimen results. There has been previous evidence that lap splice specimens are similar in bond properties to beam anchorage specimens [31,41,42,43]. As a result, the beam test database no longer distinguishes between lap splice specimens and anchorage specimens. Based on the beam test (Supplementary data) gathered from previous studies [44,45,46,47,48,49,50,51,52,53,54,55,56], the following database requirements were identified: (1) There was sufficient concrete cover on test specimens with transverse bars; (2) There was a bond failure prior to rebar yielding; and (3) Concrete cast below rebars did not cause problems with its casting position. 

The beam test database contains the test parameters for 401 beam specimens. As seen in Supplementary data: ${f_{c}}^{\prime }$ represents the compressive strength of concrete cylinders (150 mm×300 mm); $f_{y}$ refers to the yield strength of the reinforcing bars; $c_{b}$ stands for concrete bottom cover for reinforcing bars; $d_{b}$ refers to the diameter of the rebar; l represents the development length; h represents the specimen’s height; and $\tau_{u}$ is the bond stress of the reinforcing bars. It was found that transverse bars rarely yielded, and that the yield strength of transverse bars had little effect on bond strength [57].

### 3.2. Definitions for Input and Output Variables

It is necessary to identify input and output variables in order to conduct further analysis. The bond strength is predicted based on six input variables, and the bond stress is the output variable (Table 2). As noted in the references [44,45,46,47,48,49,50,51,52,53,54,55,56], these input variables have a significant impact on the bond strength of reinforcing bars. Moreover, prior knowledge should be reflected in the input variables. For instance, prior studies have demonstrated a linear relationship between ultimate bond strength and the square root of the compressive strength of concrete [9]. The bond strength of the high-performance concrete might be related to a high tensile strength [58]. The contribution of concrete to bond strength has also been described by existing formulas using the same parameters [32,33,44]. Thus, it can effectively increase the accuracy and interpretability of the model.

### 3.3. Implementation Process

**Figure 3:**
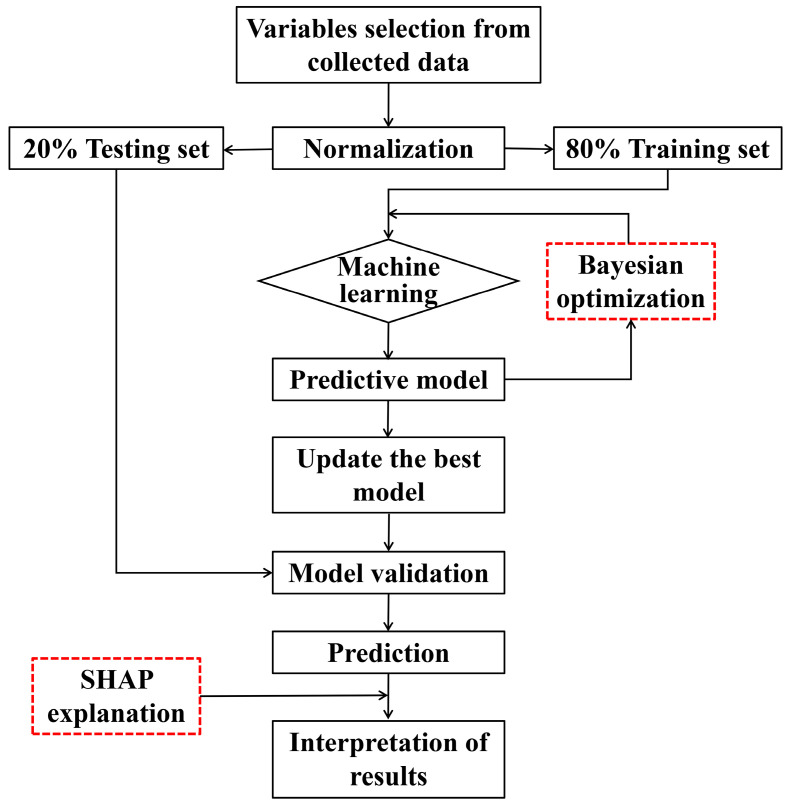
Flowchart for predicting target performance using ML algorithms.

There are generally four stages involved in the implementation of ML algorithms with Bayesian optimization (BO): As a first step, high-fidelity data must be collected and then randomly divided into two sets (80% for training and 20% for testing); As a second step, in order to reduce the error in prediction, it is recommended that a five-fold cross-validation method be used during training; In addition, the model parameters will be adjusted using Bayesian optimization; Lastly, the results of the model will be discussed using feature importance analysis through the SHAP explanation. Figure 3 illustrates how machine learning algorithms can be used to predict the performance of the target. 

For predicting bond strength ($\tau_{u}$), ML-6, an ML-based model containing six parameters, was proposed in the previous section. However, some of its parameters cannot be obtained directly from existing building structures (such as concrete cover and beam specimen height), which means that this model cannot be directly applied in practice. The study presents a simplified model (ML-3), whose input variables and output variables are shown in Table 2. 

### 3.4. Data Normalization

It is recommended that the input variables be normalized prior to training, as the extracted data have different units and ranges [59]. A similar scale is applied to all features during the normalization process. It is possible to reduce the statistical bias and improve the reliability of ML-based models by using the following normalization procedure.

(11) $$X_{i,n}=\frac{X_{i}-X_{min}}{X_{max}-X_{min}}$$

where $X_{min}$ represents the minimum value of the input variable, while $X_{max}$ represents the maximum value of the input variable.

### 3.5. K-Fold Validation

**Figure 4:**
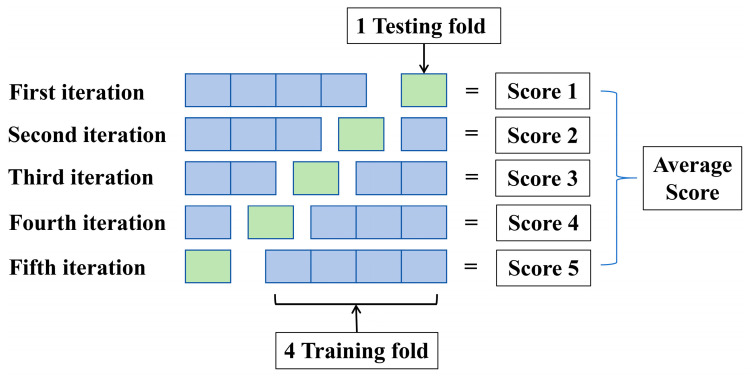
Flowchart of 5-fold cross-validation.

In the absence of an adequate dataset size, cross-validation can be used to avoid the overfitting problem. A cross-validation process involves dividing the original data into training and testing sets and reusing the data on each set. According to Figure 4, K = 5, which is referred to as 5-fold cross-validation. The following steps should be followed in detail: (1) Currently available data are divided into five equal subsets by random selection; (2) Four subsets of data are used for training, and the remaining subsets are used for testing the model; (3) It is recommended that this process be repeated five times with different folds for testing; (4) The average R square is used in this study as a criterion for evaluating the results of the model.

## 4. Results and Discussion

### 4.1. The Impact of Bayesian Optimization

**Figure 5:**
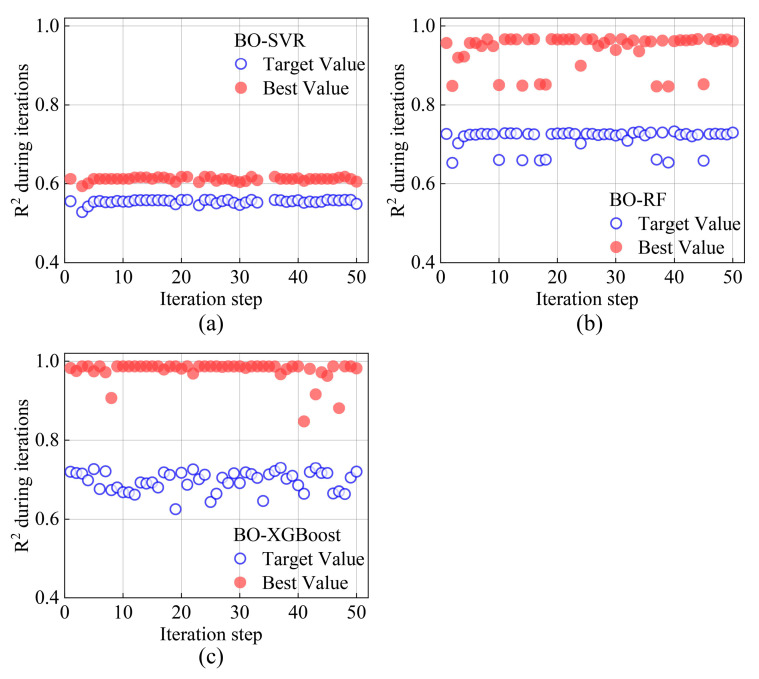
Optimizing history in model training: (**a**) BO-SVR; (**b**) BO-RF; and (**c**) BO-XGBoost.

As shown in Figure 5, the model was trained for 50 iterations before the optimal set of hyperparameters was determined. Table 3 summarizes the parameters of the ML algorithms. The objective function of Bayesian optimization is calculated based on the average R^2^ of iterations, and the following result can be obtained:

(12) $$X_{best}=\arg\max f\left(x\right)$$

Table 4 presents the final prediction results of ML-6 models in the training set and test set. Table 4 illustrates that BO-XGBoost is the most effective prediction model in both the training and test sets, with satisfied prediction results indicating adequate training for the proposed model. Based on the Bayesian optimization, the accuracy of the model is significantly improved over that of standard algorithms. Thus, optimizing the initial weights and biases using Bayesian optimization can enhance the performance of the hyperparameter determination. In addition, BO-XGBoost has developed an in-depth understanding of the relationship between influential factors and bond strength. 

### 4.2. Comparison with Empirical Models for Bond Strength

For the purpose of illustrating the advantages of ML-based models, five empirical models, including two design codes, have been introduced and their performance has been compared. Table 5 summarizes the predictions obtained from empirical models and optimized ML models for all datasets. The mean value and coefficient of variation (CoV) of the ratio between the actual value and predicted value are also reported. It is evident that ACI 408R provides the highest $R^{2}$ value (0.84), the lowest CoV (0.402), MAE (1.654 MPa) and RMSE (2.538 MPa), indicating that it is the most appropriate empirical model. This may be due to the fact that the contribution of different variables in the ACI 408R formula is more reasonable. Other formulas, for example, overestimate the contribution of the concrete cover to bond strength or underestimate the contribution of the steel rebar diameters and the compression strength of the concrete. 

The prediction performance of all ML-based models is higher than that of empirical models. The imperfect prediction performance indicates that the empirical equations may have been oversimplified and that several important factors need to be taken into account. In contrast, the BO-XGBoost model outperforms other models in terms of the highest R^2^ (0.95), lowest MAE (0.470 MPa), and RMSE (0.743 MPa), a mean value of 0.99, and the lowest CoV (0.109). 

**Figure 6:**
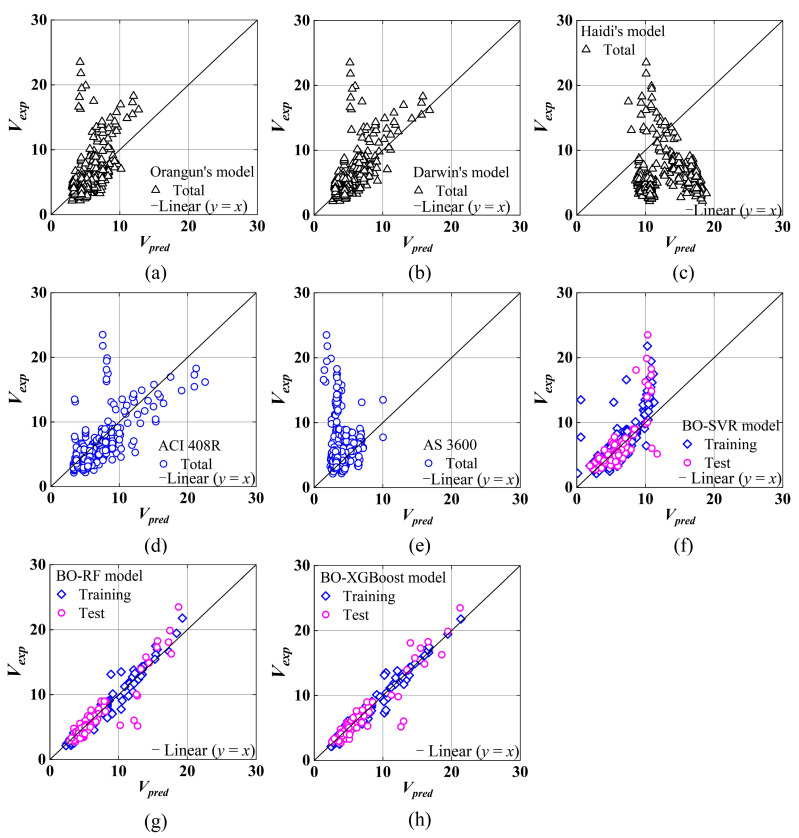
Comparison of proposed bond strength models with empirical models: (**a**) Orangun’s model; (**b**) Darwin’s model; (**c**) Haidi’s model; (**d**) ACI 408R; (**e**) AS 3600; (**f**) BO-SVR; (**g**) BO-RF; (**h**) BO-XGBoost.

It is shown in Figure 6 that the optimized ML-based models are compared with the five existing empirical models. The graph shows the correlation between predicted values and actual values. The scatter points of the BO-XGBoost model are closer to the diagonal than others. Accordingly, the BO-XGBoost model’s predicted values seem to be in better agreement with those observed.

### 4.3. Model Interpretations

#### 4.3.1. The Shapley Additive Explanation (SHAP) Theory

The Shapley additive explanation (SHAP) theory was employed in this study in order to explore the potential complexity of the nonlinear relationship between bond strength and input variables [60]. As a methodology for interpreting individual predictions, SHAP is based on the optimal Shapley value in game theory [61]. Based on this algorithm, the marginal contribution of each feature to the model output is calculated and interpreted both globally and locally by constructing an additive explanation model. In terms of the original model $f\left(x\right)$, this explanation model $g\left(x_{S}\right)$ is expressed by the following equations:

(13) $$f\left(x\right)=g\left(x_{S}\right)=\varphi_{0}+\sum_{i=1}^{N}\varphi_{i}x_{S}^{i}$$

where N refers to the number of features, x represents the input variable’s original matrix, $x_{S}$ represents the simplified matrix of input variable, $\varphi_{0}$ is defined as a constant value when there are no input variables, and the value of $\varphi_{i}$ corresponds to the Shapley value of the feature.

(14) $$\varphi^{k}\left(f,\;x\right)=\sum_{S\subseteq \left\{x^{1},\;x^{2}\cdots ,x^{N}\right\}}\frac{\left|S\right|!\left(N-\left|S\right|-1\right)!}{N!}\left(f_{x}\left(S\cup \left\{x^{N}\right\}\right)-f(S)\right)$$

where S represents the set of features that are included in model, f(S) represents the output value of a model for a particular combination of features. A summary of the SHAP theory can be found in the following reference [60,61].

#### 4.3.2. Model Interpretations for Bond Strength

While the BO-XGBoost model has excellent bond strength prediction accuracy, in general, it tends to behave like a black box, lacking adequate interpretation of factors and strength relationships. The purpose of this paper is to demonstrate the utility of the SHAP method as a tool for analyzing a complex BO-XGBoost model that has multiple input variables. 

**Figure 7:**
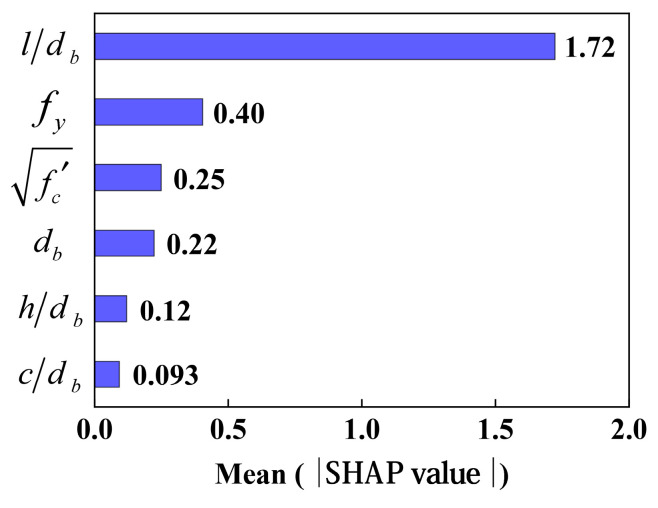
Input parameters influencing bond strength.

Based on the BO-XGBoost model, Figure 7 presents the mean SHAP value of the input variables used to predict bond strength and ranks them from high to low impact. According to Figure 7, ($l/d_{b}$) is the input variable with the highest SHAP value and is the most important component that can be used to predict bond strength. In terms of importance, ($f_{y}$) ranks second, followed by ($\sqrt{{f_{c}}^{\prime }}$), ($d_{b}$), ($h/d_{b}$) and ($c/d_{b}$). It was also noted in Refs. [20,21,24,25] that these three variables (l, $f_{y}$, $\sqrt{{f_{c}}^{\prime }}$) have a significant impact on the bond strength in physical tests.

**Figure 8:**
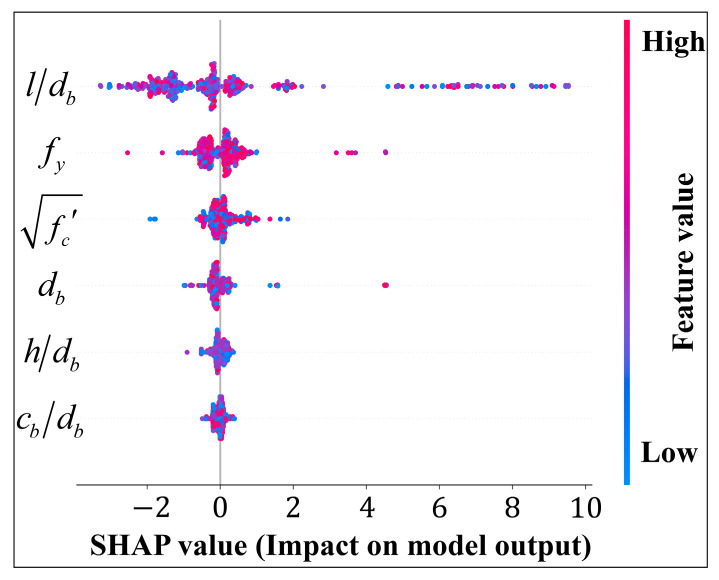
SHAP summary plot for bond strength model.

SHAP summary plots are shown in Figure 8 as a means of illustrating how input variables influence bond strength predictions. In the figure below, the SHAP value represents the contribution of each feature to the output metric. As each feature value is colored from red to blue, it indicates its size from high to low. A visual inspection of Figure 7 reveals that the bond strength increases with the increase of each of these input variables ($l/d_{b}$, $f_{y}$, $\sqrt{{f_{c}}^{\prime }}$). In contrast, the $d_{b}$ has a negative impact on the output bond strength, which suggests that the bond strength decreases as $d_{b}$ increases. 

Several studies [2,62] have investigated the effects of concrete compressive strength and rebar yield strength on bond strength. It is evident from the results that the yield strength of the rebar had a greater influence on the bond strength than the compressive strength of the concrete. The results of this study are consistent with this conclusion, and the direct contribution of these two parameters can also be seen in Figure 7 and Figure 8. In parallel, it is interesting to note that the results obtained by different researchers appear to be contradictory. It has been found by Teresa et al. [63] that an increase in rebar diameter increases bond strength, whereas Siempu et al. [64] have found that an increase in rebar diameter decreases bond strength. In spite of this, it is evident from Figure 7 and Figure 8 that the diameter of the rebar has a negative impact on bond strength. 

The influence of these two variables ($h/d_{b}$ and $c/d_{b}$) on bond strength is less than that of other input variables, and as shown in Figure 8, it is unclear whether these two parameters have a positive or negative influence. This may be due to the fact that existing research conclusions have been drawn from univariate variation results. Several factors are interconnected, and this effect of multivariate dependence can be explored further. 

### 4.4. Performance of Simplified Models (ML-3)

A simple ML model (ML-3) involving only three variables is presented in this paper as a means of obtaining bond stress in existing structures and making it easier for designers to apply it to the design process. The three input variables are $\sqrt{{f_{c}}^{\prime }}$, $f_{y}$, $d_{b}$ (Table 2), which are obtained based on the model interpretations. The selected parameters were those that have a greater impact on bond strength (Figure 7) and are relatively easy to obtain. 

The Bayesian optimization method (BO) is used in Table 6 to determine the hyperparameter values of ML-3 models. A summary of the final prediction results for the ML-3 models in the training set and test set is presented in Table 7. According to the test set, the BO-XGBoost model performed better than other models in terms of the highest $R^{2}$ (0.74), the lowest MAE (1.412 MPa), and the lowest RMSE (1.516 MPa). As compared to Table 4, the models with six parameters (ML-6) exhibit a better correlation with experimental data than simplified models (ML-3). As the number of input variables is increased, some influential factors can be taken into account that are difficult to evaluate when only three parameters ($\sqrt{{f_{c}}^{\prime }}$, $f_{y}$, $d_{b}$) are used. There is a possibility that the simplified models (ML-3) may be too global and do not completely represent the local phenomena at the interface between rebars and concrete. While both models have their advantages, they should be used in accordance with their intended purposes. A ML-6 model is more precise and conservative, but it requires six variables as inputs. Since ML-3 requires only three input variables, it is more convenient for designers to use it in practical situations. ML-3 models tend to provide better results than empirical models, except for the BO-SVR model.

### 4.5. Limitations and Future Study

In comparison to ML algorithms that require six input variables, the simplified model (ML-3) is more convenient to use. However, the results of this study indicate that the accuracy of the simplified model needs to be improved. The prediction accuracy of the simplified model needs to be improved, and future research will focus on this. Using more test data or more effective optimization parameters will enable the model to be trained more effectively. The purpose of developing a simplified machine learning model is to facilitate designers’ use of the model. Additionally, the deployment of the developed machine learning models in the form of graphical user interface tools and the creation of related design software will be beneficial to designers.

## 5. Conclusions

In this study, a data-driven approach is proposed for estimating the bond strength between steel rebars and concrete. A total of 401 beam tests have been collected and used for the validation of the proposed ML models. The accuracy of existing bond strength equations revealed significant dispersion and a lack of precision when applied to the collected data. To predict bond strength, three ML algorithms were used: SVR, RF, and XGBoost. A Bayesian optimization approach was used to enhance the performance of the model. In order to make it more convenient to apply, a simplified model (ML-3) was proposed. Conclusions were drawn as follows:

(1) Empirical models have a low prediction accuracy for the experimental data collected, and the scatter between the prediction and measurement shows the inherent difficulty of conventional explicit approaches to bond strength estimation.
(2) As a result of adequate training, BO-XGBoost proved to be the most effective prediction model in both training and test sets.
(3) With the increase of each of these three input variables ($l/d_{b}$, $f_{y}$, $\sqrt{{f_{c}}^{\prime }}$), the bond strength increases, while the $d_{b}$ has a negative impact on it.
(4) It is unclear how ($h/d_{b}$) and ($c/d_{b}$) affect bond strength. It is possible that several factors are interconnected in predicted models, which should be explored further.
(5) Both models have advantages, however, and should be utilized appropriately. A ML-6 model is more precise and conservative, but it requires six variables as inputs. Since ML-3 requires only three input variables, it is more convenient for designers to use it in practical situations.

## Figures and Tables

**Table 1:** Existing bond strength equations.

Authors	Equations
Orangun et al. [9]	$\tau_{u}=0.083045\sqrt{{f_{c}}^{\prime }}[1.2+3\left(c/d_{b}\right)+50\left(d_{b}/L\right)]$
Darwin et al. [31]	$\tau_{u}=0.083045\sqrt{{f_{c}}^{\prime }}\left[\left(1.06+2.12\left(c/d_{b}\right)\right)\left(0.92+0.08\left(c_{max}/c_{min}\right)\right)+75\left(d_{b}/c_{min}\right)\right]$
Haidi [33]	$\tau_{u}=0.083045\sqrt{{f_{c}}^{\prime }}[22.8-0.208\left(c/d_{b}\right)-38.212\left(d_{b}/L\right)]$
ACI 408R-03 [1]	$\frac{\tau_{u}}{\sqrt{{f_{c}}^{\prime }}}=0.33+0.025\left(c/d_{b}\right)+8.3\left(d_{b}/L\right)$
AS 3600 [32]	$\tau_{u}=0.265\sqrt{{f_{c}}^{\prime }}[0.5+\left(c/d_{b}\right)]$

Note: ${f_{c}}^{\prime }$ represents the cylinder compressive strength of concrete in MPa, *c* represents the front of the concrete that has the least thickness in mm, $d_{b}$ refers to the diameter of the reinforcing bar, and *L* indicates the embedded length.

**Table 2:** Summary information on the variables.

Notation	Unit	Variables	Types	
			ML-6	ML-3
$\sqrt{{f_{c}}^{\prime }}$	-	Square root of the compressive strength of concrete	Input	Input
$f_{y}$	MPa	Yield strength of the reinforcing bars	Input	Input
$d_{b}$	mm	Diameter of the rebars	Input	Input
$c_{b}/d_{b}$	-	Concrete cover to rebar diameter ratio	Input	-
$l/d_{b}$	-	Development length to rebar diameter ratio	Input	-
$h/d_{b}$	-	Height of specimen to rebar diameter ratio	Input	-
$\tau_{u}$	MPa	Bond stress	Output	Output

**Table 3:** The main parameters of comparison models (ML-6).

Algorithm	Initial Basic Parameters	After Bayesian Optimization
SVR	C = 1; gamma = 1; kernel’linear’; degree = 3; coef0 = 0; tolerance = 1 × 10^−3^; C = 1; epsilon = 0.1; shrinking = True.	C = 11; gamma = 2; kernel’linear’; degree = 3; coef0 = 0; tolerance = 1 × 10^−3^; C = 1; epsilon = 0.1; shrinking = True.
RF	n of estimators = 30; max depth = 3; criterion = ’squared error’; min samples split = 2; min samples leaf = 1; random state = 1	n of estimators = 52; max depth = 10; criterion = ’squared error’; min samples split = 2; min samples leaf = 1; random state = 1.
XGBoost	n estimators = 30; learning rate = 0.1; max depth = 3; objective = ’linear’; booster = ’gbtree’; min child weight = 1; subsample = 1; colsample bytree = 1; alpha = 0; lambda = 1.	n estimators = 99; learning rate = 0.1121; max depth = 4; objective = ’linear’; booster = ’gbtree’; min child weight = 1; subsample = 1; colsample bytree = 1; alpha = 0; lambda = 1.

**Table 4:** Summary of prediction results in training set and test set.

Proposed Models	Training Set			Test Set		
	R^2^	MAE (MPa)	RMSE (MPa)	R^2^	MAE (MPa)	RMSE (MPa)
SVR	0.54	1.203	2.073	0.44	1.755	3.189
RF	0.85	0.882	1.191	0.79	1.231	1.949
XGBoost	0.90	0.710	0.977	0.81	1.160	1.865
BO-SVR	0.61	1.132	1.896	0.52	1.715	2.960
BO-RF	0.96	0.367	0.595	0.85	0.947	1.621
BO-XGBoost	0.97	0.364	0.550	0.87	0.897	1.516

**Table 5:** Summary of bond strength prediction in all datasets.

Model	$\mathrm{Mean}\;(V_{n,exp}/V_{n,pred}$)	$\mathrm{CoV}\;(V_{n,exp}/V_{n,pred}$)	$R^{2}$	MAE (MPa)	RMSE (MPa)
Orangun [9]	1.128	0.502	0.62	1.742	2.896
Darwin [31]	1.125	0.403	0.72	1.506	2.573
Haidi [33]	0.517	0.633	0.59	7.068	7.858
ACI 408R-03 [1]	0.971	0.402	0.84	1.654	2.538
AS 3600 [32]	1.694	0.902	0.60	2.400	4.158
BO-SVR	1.161	1.268	0.60	1.249	2.109
BO-RF	0.993	0.109	0.94	0.483	0.800
BO-XGBoost	0.994	0.109	0.95	0.470	0.743

**Table 6:** Values of hyperparameters of ML-3 models.

Proposed Models	Value of Hyperparameters
BO-SVR	C = 15; gamma = 13; kernel’linear’; degree = 3; coef0 = 0; tolerance = 1 × 10^−3^; C = 1; epsilon = 0.1; shrinking = True.
BO-RF	n of estimators = 105; max depth = 6; criterion = ’squared error’; min samples split = 2; min samples leaf = 1; random state = 1.
BO-XGBoost	n estimators = 32; learning rate = 0.2034; max depth = 3; objective = ’linear’; booster = ’gbtree’; min child weight = 1; subsample = 1; colsample bytree = 1; alpha = 0; lambda = 1.

**Table 7:** Summary of ML-3 prediction results.

Proposed Models	Training Set			Test Set		
	R^2^	MAE (MPa)	RMSE (MPa)	R^2^	MAE (MPa)	RMSE (MPa)
BO-SVR	0.25	1.695	2.644	0.15	2.296	3.941
BO-RF	0.86	0.852	1.113	0.70	1.450	2.347
BO-XGBoost	0.85	0.910	1.196	0.74	1.412	1.516

## Data Availability

The data presented in this study are available on request from the corresponding author due to privacy.

## Supplementary Materials

The following supporting information can be downloaded at: https://www.mdpi.com/article/10.3390/ma17184641/s1, supplementary data and code of manuscript.
